# Dental Periodicals

**Published:** 1862-01

**Authors:** 


*Dental periodicals*, next to colleges, claim some attention as being the vehicle in the dissemination of useful professional knowledge. It is therefore all important that the matter they contain shall unmistakably tend to the accomplishment of this purpose; but how much do we find in them to condemn that is either prejudicial to the character of the profession, a positive injury to the student, or a sinful waste of paper and ink and of the time of the reader. As an illustration let any one take the November number of the "Vulcanite," and turn to page 131, and read the article with the greatest care, and then turn to and read other communications, from the same pen in other dental periodicals, and I think he will agree with me, that either the publishers of these journals are in great want of matter, or that their kind feelings to the writer of them, (in which I claim to participate) or regard to the position he has occupied in the profession, prevents their declining his communications. *That he is in terrible earnest, and purely desirous of rendering all possible service to the profession, I religiously believe;* but I submit do such productions result in any thing more enduring than a smile, and is it the best use that can be made of the pages of our journals?

Let me give one or more brief extracts. From the journal already named I take the following; the subject under discussion, it will be noticed, is "professional standing:"

"In nature the ethereal vastness between the planetary bodies is one grand field of perfectly harmonious relations. The upper currents of atmosphere become more and more harmonious as they ascend to the thinner, purer, and higher strata on the periphery of the atmospheric zone; while the violence of contending currents, constituting winds and storms, increase as they approach the surface of the ponderable and immovable 'conservative' earth's crust; and though unseen by the eye, these currents gather force as they advance, till finally everything ponderable and massive becomes as nothing in their destructive, devastating march; and then, we plainly see, hear, and feel the effects that depend upon this unseen and to some minds incomprehensible power."

What relation all this vaporing has to the subject under discussion, I leave for the reader to discover. I don't see it.

Again: In the Journal, published in this city, November number, page 180, (no matter what the subject, the same drift or vein is still worked,) in alluding to a spiritual organism, we have the following, "aspirations of a higher life, making us willing to pass cheerfully the cataclysms of the morphological lave." * * * "Our electrospheres send out emanations like rays of light to the vastness of space, and communicate appulse to other electrospheres of like tenuity, thus establishing a sort of commerce between them, that may, or may not be conscious, to the individuals exerting the influence."

But it were sheer folly and a waste of time to go any further with these extracts, for all his papers abound in just such jargon. Such matter would be eminently in place in the "Spiritual Advocate," but sadly out of place in a dental Journal, and its admission is a perversion of the editorial license.

Speaking of dental journals, I want to say what may be news to many in the profession, which is, that I never knew of but one dental periodical that paid more than the printers bill from its receipts, and that one died; not, as one might suppose from joy at its success, but because of the decease of its senior editor, the lamented Harris: and I have known of but one other that ever paid the printer from its subscriptions.

Now these facts are conclusive evidence of the criminal neglect of our journals by the profession, and it is to me painfully humiliating. Were it not for other interests the publication of all but one of our present journals serve, in being connected with dental depots, etc., we should have but a very limited number, perhaps but one; for the simple reason that they are not self-sustaining; and I very much doubt whether a publication devoted exclusively to the interests of the profession, enlisting the best talent, and conducted with spirit and ability, depending solely upon its subscription list for support, could more than pay the printer's bill.

These are unpleasant truths, and it is quite time they were generally known, and they should impress themselves upon the minds and consciences of the profession.

Yours, O. U. C.

*Philadelphia, January*, 1862.

